# Kinetic and Structural Determinants for GABA-A Receptor Potentiation by Neuroactive Steroids

**DOI:** 10.2174/157015910790909458

**Published:** 2010-03

**Authors:** Gustav Akk, Douglas F. Covey, Alex S. Evers, Steven Mennerick, Charles F. Zorumski, Joe Henry Steinbach

**Affiliations:** Departments of Anesthesiology (GA, ASE, JHS), Developmental Biology (DFC), and Psychiatry (SM, CFZ), Washington University School of Medicine, St. Louis, MO, USA

**Keywords:** Receptor, channel, patch clamp, kinetics.

## Abstract

Endogenous neurosteroids and synthetic neuroactive steroid analogs are among the most potent and efficacious potentiators of the mammalian GABA-A receptor. The compounds interact with one or more sites on the receptor leading to an increase in the channel open probability through a set of changes in the open and closed time distributions. The endogenous neurosteroid allopregnanolone potentiates the α1β2γ2L GABA-A receptor by enhancing the mean duration and prevalence of the longest-lived open time component and by reducing the prevalence of the longest-lived intracluster closed time component. Thus the channel mean open time is increased and the mean closed time duration is decreased, resulting in potentiation of channel function. Some of the other previously characterized neurosteroids and steroid analogs act through similar mechanisms while others affect a subset of these parameters. The steroids modulate the GABA-A receptor through interactions with the membrane-spanning region of the receptor. However, the number of binding sites that mediate the actions of steroids is unclear. We discuss data supporting the notions of a single site vs. multiple sites mediating the potentiating actions of steroids.

## INTRODUCTION

Neurosteroids are synthesized in the central and peripheral nervous system from cholesterol or other steroidal precursors [9, 14, 31]. The enzymes required for neurosteroidogenesis are present in oligodendrocytes and astrocytes as well as at least some types of neurons [29, 38, 41]. The neurosteroids synthesized in and secreted from glial cells act as paracrine messengers. In the case of neuronal neurosteroidogenesis the neurosteroids may act locally on their targets through an autocrine mechanism [1].

From a viewpoint of medicinal chemistry, the steroid ring structure serves as a useful pharmacophore template to create synthetic neuroactive steroid analogs. There are two general motivations for such work. First, studies on structure-activity relationships can yield novel information on the function and modulation of the target receptor, and second, the studies can help generate receptor subtype-specific compounds with therapeutic potential. Indeed, ongoing interest in the study and development of potentiating steroids is driven by the behavioral effects of endogenous steroids and the potential therapeutic use of exogenous steroid analogs [17, 30].

	Neurosteroids and synthetic steroid analogs have multiple targets in the brain. These include transmitter-gated ion channels such as the GABA-A and NMDA receptors [28, 39]. Steroids can also modulate some voltage-gated calcium channels thereby modifying transmitter release [18] or altering pain threshold in animal models [37]. Other cellular processes modulated by neurosteroids are microtubule assembly affecting neural cytoskeleton dynamics [16] and regulation of cell death [12].

The GABA-A receptor is an anion-selective pentameric receptor-channel whose activation in most cases has an inhibitory influence on the cell. Accordingly, drugs capable of potentiating the GABA-A receptor activity can be useful as anesthetics, anticonvulsants and anxiolytics. Many neurosteroids are potent and efficacious modulators of the GABA-A receptor. It is likely that GABA-A receptors *in vivo* are under tonic, but submaximal neurosteroid modulatory control [10, 26]. The nature of modulation can be inhibitory or potentiating, depending on the structure of the steroid. Typically, steroids containing charged groups (e.g., pregnenolone sulfate) act as inhibitors of the GABA-A receptor. Most potentiating steroids also directly activate the GABA-A receptor when used at high concentrations. It is likely that inhibition, potentiation, and direct activation are mediated by steroid interactions with distinct sites [3, 20, 21, 33, 34]. In this review, we discuss the kinetic and structural features underlying the rat α1β2γ2L GABA-A receptor potentiation by endogenous neurosteroids and synthetic steroid analogs. We focus on the published work from our laboratories and introduce some novel, previously unpublished data.

## KINETIC MECHANISMS OF ACTION OF POTENTIATING STEROIDS

**Fig. (1):**
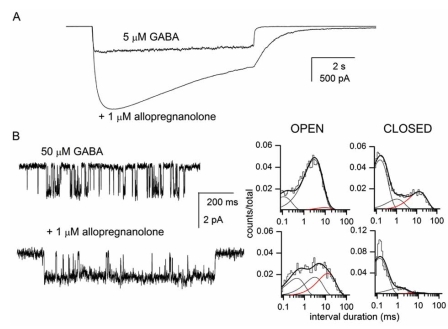
**Potentiation of the α1β2γ2L GABA-A receptor by allopregnanolone. (A)** Whole-cell recordings from a HEK cell expressing wild-type α1β2γ2L GABA-A receptors. The cell was exposed to 5 µM GABA in the absence and presence of 1 µM allopregnanolone. 5 µM GABA corresponds to approximately EC20 in the activation concentration-effect curve. 1 µM allopregnanolone corresponds to a maximally potentiating concentration. **(B)** Sample single-channel currents from cell-attached patches exposed to 50 µM GABA (top trace) or GABA + 1 µM allopregnanolone (bottom trace). Openings are shown as downward deflections. The open and closed time histograms from the respective patches are shown next to the data traces. Only single-channel clusters, i.e., data resulting from the activation of a single receptorchannel, were used in the analysis. Allopregnanolone potentiates the α1β2γ2L GABA-A receptor by enhancing the mean duration and prevalence of the longest of the three open time components (red line in open time histograms) and reducing the prevalence of the longest of the three intracluster closed time components (red line in closed time histograms). For GABA, the open times were 0.11 ms (21 %), 2.8 ms (75 %) and 8 ms (4 %), and the closed times were 0.14 ms (64 %), 1 ms (13 %) and 12 ms (22 %). For GABA + allopregnanolone, the open times were 0.39 ms (32 %), 2.8 ms (32 %) and 12 ms (35 %), and the closed times were 0.12 ms (83 %), 1 ms (14 %) and 10 ms (3 %). The data shown in the figure have not been published previously.

In whole-cell recordings, coapplication of potentiating neurosteroids with agonist leads to enhancement of the peak response (Fig. **1A**). The magnitude of potentiation depends on the concentration of agonist used to activate the receptors, the effect is greater in the presence of lower concentrations of agonist. At 5 μM GABA (~EC_20_), exposure of α1β2γ2L GABA-A receptors expressed in human embryonic kidney cells to high concentrations of neurosteroids such as allopregnanolone elicits an approximately 3-4-fold potentiation of peak response [26].

Single-channel currents recorded at high GABA concentrations (EC_20_ and above) exhibit characteristic "single-channel clusters", i.e., episodes of high-frequency activity originating from a single receptor [25, 36]. Clusters contain a wealth of information about the activation properties of the receptor; most importantly, the kinetic studies on single-channel clusters allow one to focus on the actions of the modulator on a single receptor and eliminate the ambiguity about changes in the number of active receptors or single-channel conductance.

The open and closed time histograms generated from single-channel clusters from the α1β2γ2L GABA-A receptor each contain three components (e.g., [36]). The open time distributions are generally not affected by changes in GABA concentration indicating that the majority of openings at these GABA concentrations (i.e., above EC_20_) originate from fully-liganded receptors. The mean durations and prevalence of two of the closed time components remain unchanged throughout a wide range of GABA concentrations. We previously proposed that the states producing these closed time components are not on the return pathway to the resting receptor [36]. The mean duration of the third component in the closed time histograms scales with agonist concentration (it is briefer when higher concentrations of GABA are used to activate the receptor). This closed time component likely reflects steps in the channel activation pathway, i.e., transitions from the unliganded, closed state to the fully-liganded open state.

Work on GABA-A receptor single-channel activity has shown that steroid-elicited potentiation results from enhanced open probability (Po) of the receptor, not changes in single-channel conductance or the number of active receptors. The increase in open probability in the presence of potentiating steroids results from specific changes in the channel open and closed time distributions (Fig. **1B**). Overall, the mean open duration of the channel is increased and the mean closed time duration is decreased. The prolongation of the mean open time results from two specific changes in receptor kinetics. First, the mean duration of the longest of the three open time components (OT3) is increased. In the presence of GABA alone, the mean duration of OT3 is 6-7 ms [26, 36]. When high concentrations of potentiating steroids are coapplied with GABA the mean duration of OT3 is increased to 15-25 ms [3, 7]. Second, the relative frequency of OT3 increases from approximately 15 % to over 30 % at high concentrations of steroids, i.e., the long openings become more prevalent. The reduction in the mean intracluster closed time duration in the presence of potentiating steroids is the result of a selective reduction in the prevalence of the longest-lived closed time component (CT3). The prevalence of CT3 is approximately 25 % in the presence of GABA [36]. This value is reduced to 3-10 % in the presence of potentiating steroids [3, 23].

All three kinetic effects contribute to the overall potentiating effect, however the relative contributions are not equal. Fig. (**2**) shows the calculated contributions from the individual components to the total, overall potentiation. The calculations are based on our single-channel work on the rat α1β2γ2L GABA-A receptor [3, 36] and were carried out at a GABA concentration producing 20 % of maximal response. The range of steroid effects on the open and closed times was held to that measured for allopregnanolone [26]. According to the calculations the peak response at 1 μM steroid is 340 % of control (no effect equals 100 %). This value is similar to the experimental findings on whole-cell potentiation by allopregnanolone (range of 346 - 438 %; [25, 26]. The graph indicates that the greatest contribution to total potentiation is made by the steroid effect on the closed time distribution. If only this parameter was modified by the steroid, the relative response would be 238 % of control. Steroid effects on the mean duration and prevalence of OT3 make smaller contributions to the overall potentiation. Receptor responses would be 121 and 126 % of control if the steroid affected solely the mean duration or prevalence of the longest-lived open time component, respectively.

The pattern of three kinetic effects triggered by steroids as described above is typical for many neuroactive steroids including allopregnanolone [7], pregnanolone [24], and the synthetic steroid analog (3α,5α,17β)-3-hydroxyandrostane-17-carbonitrile [3]. On the other hand, some neuroactive steroids selectively affect a subset of kinetic parameters. For example, the endogenous steroid etiocholanolone increases the prevalence of long openings without affecting the mean duration of long openings or the closed time distributions [24]. As expected based on the analysis presented in Fig. (**2**), the overall potentiating effect of etiocholanolone as measured in whole-cell recordings is relatively weak [22, 24]. Furthermore, cyclopenta[*b*]phenanthrenes and cyclopenta[*b*] anthracenes, tetracyclic molecules with ring systems different from that of steroids, have been shown to selectively modify receptor kinetics [33], but the relationship between the ring system and kinetic parameters affected by the drug is unclear at the moment.

Despite extensive kinetic studies, relatively little is known about which molecular processes or kinetic rate constants are affected by steroids. In part this is due to the complex kinetic properties of the GABA-A receptor and the lack of a commonly accepted kinetic model for its activation. Nevertheless, several conclusions regarding the mechanisms of steroid actions can be drawn, even in the absence of a specific kinetic model. For example, we suggest that potentiating steroids do not affect receptor affinity to GABA or the channel opening rate constant. This conclusion is based on the finding that the channel effective opening rate curve is unaltered in the presence of steroids [3]. The effective opening rate, which is defined as an inverse of the agonist concentration-dependent closed time component (please see above), is dependent on receptor affinity to the agonist and the channel opening rate constant in the presence of a given agonist. Identical effective opening rate curves are thus an indication of identical affinity and channel opening properties.

The extent of receptor potentiation depends on the concentration of GABA at which the effect is measured. In general, potentiation caused by changes in kinetics is limited to an open probability of 1. The α1β2γ2L GABA-A receptor has a maximal single-channel open probability of approximately 0.9, hence little potentiation can be seen at high or saturating concentrations of GABA. In the presence of a concentration of GABA eliciting 20 % of maximal response, neurosteroids such as allopregnanolone can produce 3-4-fold potentiation of the peak whole-cell response. At lower concentrations of GABA the degree of potentiation increases due to expanded dynamic range (e.g., [11]). For example, when 0.5 μM GABA (~EC_5_) is used to activate the receptor potentiation by saturating concentrations of steroids can reach 20-fold [24].

GABA-A receptors containing the δ subunit are considered to be highly sensitive to potentiating steroids. The underlying reason for this does not lie in the improved interactions of steroids with such receptors. Rather it is caused by the low gating efficacy of the δ subunit-containing receptor. The α1β2δ and α4β2δ receptors are only weakly activated by GABA. The maximal open probability of these receptors in the presence of GABA is very low, probably <0.1 [2, 15, 40]. As a result, the dynamic range for potentiation is enhanced. These receptors appear to be more strongly modulated by steroids and can show potentiation even when exposed to saturating GABA concentrations.

## LOCATION OF BINDING SITES FOR POTENTIATING STEROIDS

Work from several laboratories has led to a conclusion that the binding sites for potentiating steroids are located in the membrane-spanning regions of the GABA-A receptor [7, 19, 26, 32]. By employing single-channel patch clamp recordings we showed that receptors in cells pretreated with a neuroactive steroid that was subsequently removed from the bath remained potentiated for long periods of time. Furthermore, receptors in patches excised from such cells remained potentiated suggesting that the steroid accumulated in the cell membrane and was capable of eliciting potentiation even after patch excision [7]. In macroscopic whole-cell recordings the effect manifests as slow deactivation upon the removal of the steroid and agonist from the bath.

To exclude the possibility that the slow washout of steroid effect results from slow unbinding of the steroid from the receptor, we examined the effect of cyclodextrins on the deactivation rate. Cyclodextrins are membrane-impermeant cyclic sugar molecules that can act as steroid scavengers by forming an inclusion complex with the steroid [27, 35]. Thus, cyclodextrins can be used to reduce the concentration of free steroid in the membrane. We found that the inclusion of cyclodextrins in the bath during the washout phase drastically enhanced the deactivation rate [7, 13, 35]. This indicates that the long-lasting potentiating effect of steroids is dominated not by their slow dissociation from the receptor but rather by the retention of steroids in the membrane where they presumably act locally on the receptor.

By exploiting the difference in the ability of steroids to potentiate insect and mammalian GABA receptors, Hosie and coworkers [20] showed that steroid sensitivity correlated with the structures of the M1 and M4 transmembrane domains of the α subunit. A chimeric approach followed by site-directed mutagenesis of residues in these regions helped to identify specific residues that were proposed to interact *via *hydrogen bonding with the C3 hydroxyl (α1Q241 in M1) and C20 ketone groups (α1N407 and Y410 in M4) of the neurosteroid allopregnanolone. Mutations eliminating the hydrogen bonding capability of these residues drastically reduced or abolished potentiation by several potentiating neurosteroids without affecting potentiation by other classes of potentiators such as barbiturates or benzodiazepines [20].

## HOW MANY SITES ARE ON THE GABA-A RECEPTOR FOR POTENTIATING STEROIDS?

The number of interaction sites mediating GABA-A receptor potentiation by neuroactive steroids is not entirely clear at present. Although steroids can have multiple kinetic effects on channel function, the effects do not necessarily have to be mediated by distinct sites. A single interaction site could mediate multiple kinetic actions. The electrophysiological and molecular biological approaches have yielded conflicting evidence, some of which support the notion of a single interaction sites for steroids. Other data are best accounted for by the presence of multiple, non-overlapping sites mediating the various potentiating effects of steroids. Here, we briefly describe the evidence supporting the two notions.

The strongest support for a single interaction site mediating the potentiating actions of steroids comes from mutagenesis experiments. Such studies have shown that mutation of the α1Q241 residue can affect all kinetic aspects of potentiation by neurosteroids. For example, a receptor containing the α1Q241L mutation shows none of the kinetic effects on open and closed time distributions seen in the wild-type receptor in the presence of the neurosteroid allopregnanolone [4]. In the binding site model proposed by Hosie *et al*. [20] the α1Q241 residue forms a critical hydrogen bond with the C3 hydroxyl group. Mutant receptors incapable of forming a hydrogen bond (e.g., α1Q241L) are therefore predicted to lack all kinetic components if a single site, one defined by the α1Q241 residue, mediates potentiation.

The second piece of evidence supporting a single binding site for potentiating steroids comes from our work with the synthetic steroid analog (3α,5α,11β)-3-hydroxy-11-methyl-11-[[4-[3-(trifluoromethyl)-3H-diazirin-3-yl]phenyl]methoxy]-pregnan-20-one (Fig. **3**; 11-TFMPD3α5αP). A coapplication of 11-TFMPD3α5αP with GABA modifies the open and closed time distributions not unlike allopregnanolone indicating that the synthetic steroid acts on the same kinetic processes. An examination of single-channel activity demonstrates that in the presence of steroid, channel activity consists of long-lived alternating periods of low and high open probability (Fig. **3A**). The two modes of activity resemble channel activity observed under control conditions (in the presence of GABA alone) and in the presence of GABA and a high dose of potentiating steroid such as allopregnanolone. Further investigation revealed that the mean duration of episodes with low open probability depends on the concentration of 11-TFMPD3α5αP. The low Po episodes become shorter as the concentration of steroid is increased (Fig. **3B**). In contrast, the mean duration of high Po episodes remained essentially unchanged at all steroid concentrations. The findings could be explained by a simple model where steroid binding to the receptor enhances channel Po through a binary mode switch, and the high and low Po episodes reflect activity with and without associated steroid, respectively.

The transitions between low and high Po episodes can provide insight into steroid interaction with the receptor. If the individual kinetic effects of the steroid result from independent interaction with multiple distinct sites, then the data should consist of episodes in which any number of the parameters is modified. For example, we should be able to detect episodes in which the closed time distributions are modified but open time distributions remain like those in the presence of GABA alone. On the other hand, if steroid interaction with a single site mediates all changes in open and closed time distributions, then the binding of steroid to the receptor would modify all parameters. The data presented in Table **1** indicate that within episodes of high open probability all three kinetic parameters contributing to potentiation were modified. In contrast, within episodes of low open probability none of the three kinetic parameters was significantly different from that in the presence of GABA alone. Given the low probability of simultaneous, concerted binding to three distinct, independent steroid binding sites, the data appear to suggest that the binding of a single steroid molecule elicits all three kinetic changes.

There are also several findings that cannot be easily explained in the framework of a single interaction site. The most striking is the results from the experiments examining wild-type GABA-A receptor potentiation in the simultaneous presence of pregnanolone and etiocholanolone. The two steroids differ in their kinetic mechanisms of action. Pregnanolone acts by enhancing the mean duration and prevalence of the long-lived open time component and decreasing the prevalence of long intracluster closed times, i.e., similar to allopregnanolone. Etiocholanolone only affects the prevalence of long openings [24]. The difference could be accounted for by dissimilar interactions of the two steroids with the receptor within a single, common binding site, or by the steroids interacting with different subsets of three separate sites. To attempt to distinguish between the two mechanisms, we examined the effect of pregnanolone in the presence of etiocholanolone. We reasoned that if a single site mediates all actions of the two steroids then the presence of etiocholanolone, a steroid with a single kinetic effect, would reduce the ability of pregnanolone to elicit a change in the mean duration of OT3 and the prevalence of CT3. In essence, etiocholanolone would act as a competitive inhibitor in this scenario. On the other hand, if the kinetic effects are mediated by steroid interactions with distinct, non-overlapping sites then etiocholanolone should have no effect on the ability of pregnanolone to elicit said changes. The single-channel recordings unequivocally demonstrate that etiocholanolone has no effect on the ability of pregnanolone to potentiate the receptor [24]. In particular, the effect of pregnanolone on the mean duration of OT3 and the prevalence of CT3 is unchanged in the presence of etiocholanolone. These findings are most consistent with the concept of multiple, non-overlapping steroid binding sites mediating receptor potentiation [24].

Additional evidence for the presence of multiple binding sites comes from the comparison of the concentration-effect relationships for the kinetic effects. A single site would be most compatible with identical concentration-effect curves describing the kinetic effects. However, the data show that the synthetic neuroactive steroid (3α,5β,17β)-3-hydroxy-18-norandrostane-17-carbonitrile has considerably different potencies for the kinetic effects. The steroid modulates the prevalence of long openings and long-lived closed times with EC_50_-s at approximately 20 nM. In contrast, the EC_50_ for the change in the mean duration of long-lived openings is >10 μM [3].

We have previously shown that coapplication of ethanol can affect the ability of steroids to potentiate the α1β2γ2L GABA-A receptor [6, 8]. Interestingly, ethanol only affects changes in one of the kinetic parameters affected by steroids. The concentration-effect relationship for the allopregnanolone-induced increase in the mean duration of OT3 is shifted in the presence of 0.1 % ethanol by over 100-fold to lower steroid concentrations. The concentration-effect relationships for other kinetic parameters were found to be unaffected [6]. In the model of multiple binding sites mediating steroid effects on the GABA-A receptor, these findings suggest that ethanol selectively modulates the interaction of allopregnanolone with the site that mediates the increase in the mean duration of long openings. But we note that a model in which steroids interact with a single site while ethanol selectively modulates the gating / signal transduction element for one of the kinetic effects could also account for the findings.

The GABA-A receptor contains two α subunits and hence two steroid sites as defined by the α1Q241 residue [20]. It could be hypothesized that the sites on the two α subunits mediate distinct kinetic effects, e.g., steroid interaction with one of the α subunits could lead to the effect on closed times, the other mediates the effects on open times. We tested this hypothesis by employing concatemeric receptors, i.e., receptors in which two or more subunits are covalently linked to each other by short linkers. We generated a γβα triple subunit and a βα tandem subunit. The Q241L mutation was then selectively introduced to either of the two α subunits, and the ability of allopregnanolone to potentiate the mutated concatemeric receptor probed.

**Fig. (2):**
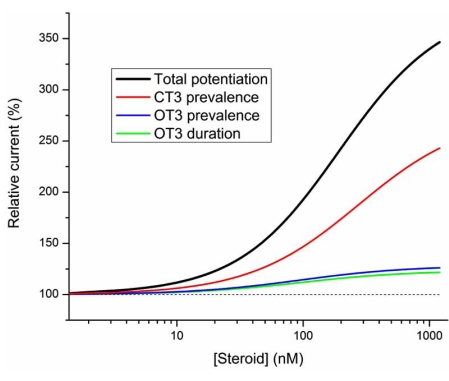
**PTotal potentiation by steroids is dominated by the effect on the prevalence of long intracluster closed times.** The curves show total potentiation (black line) and potentiation under conditions where only the duration (green) or prevalence of long openings (blue), or the prevalence of long closed times (red) is affected by the steroid. The calculations were carried out based on the Po and effective opening rate data from the rat α1β2γ2L receptor expressed in HEK cells [33]. The dashed line represents control response. The steroid effect was calculated in the presence of EC20 GABA, and the extent of steroid effect on each of the kinetic parameters was held similar to that seen in the presence of 1 µM allopregnanolone [24]. The mean duration of OT3 was 7.3 ms in the absence of steroid and 14.1 ms in the presence of a saturating concentration of steroid. The prevalence of OT3 was 13 % in the absence of steroid and 38 % in the presence of a saturating concentration of steroid. The prevalence of CT3 was 27 % in the absence of steroid and 5 % in the presence of a saturating concentration of steroid. The EC_50_-s for each of the kinetic effects of the steroid were held at 100 nM. The data shown in the figure have not been published previously.

**Fig. (3):**
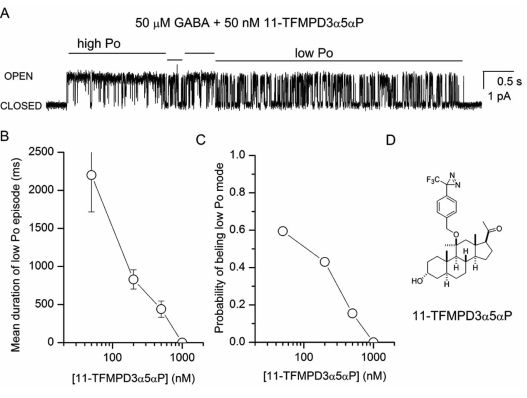
**Mode switching in the presence of 11-TFMPD3α5αP. (A)** A sample single-channel cluster from a cell-attached patch exposed to 50 µM GABA + 50 nM 11-TFMPD3α5αP. For illustrative purposes channel openings are shown as upward deflections. The locations of mode switches between episodes of high and low open probability (Po) were selected by eye. **(B)** The mean duration of low Po episodes decreases as the steroid concentration is increased. **(C)** The probability of a receptor being in the low Po mode is reduced at higher steroid concentrations. The parameter was calculated as the mean fraction of time spent in the low Po mode. **(D)** Structure of the steroid analog 11- TFMPD3α5αP. The data shown in the figure have not been published previously.

In macroscopic recordings, receptors containing the α1Q241L mutation in both the γβα and βα constructs were insensitive to allopregnanolone. When only one of the two α subunits contained the mutation (as in γβαQ241L - βα or γβα - βαQ241L receptors), allopregnanolone remained capable of eliciting potentiation, albeit with somewhat lower potency [5, 11]. Notably, the steroid concentration-effect relationships were indistinguishable for the two types of concatemeric receptors containing a single mutated α subunit. Given the strong dependence of the degree of observed potentiation on the nature of the kinetic effects mediating potentiation (please see Fig. **2**), it appeared likely that similar kinetic mechanisms underlie potentiation in both mutant concatemers.

We were able to conduct single-channel recordings on the concatemeric receptor containing the α1Q241L mutation in the γβα construct (the expression level of γβα - βαQ241L receptors was too low for single-channel studies). The data indicate that the kinetic mechanisms of potentiation are similar in the wild-type concatemeric receptor and in the concatemeric receptor containing the α1Q241L mutation in the γβα construct. The coapplication of allopregnanolone with GABA resulted in an increase in the mean duration and prevalence of long openings and a reduction in the prevalence of long closed times [5]. The data thus indicate that steroid interactions with a single α subunit can lead to changes in the open and closed time distributions and mediate potentiation by neuroactive steroids.

## SUMMARY

In sum, the electrophysiological data indicate that potentiating steroids act on the GABA-A receptor through specific changes in channel open and closed time properties. Many steroids, including the endogenous neurosteroid allopregnanolone act by modulating a total of three kinetic parameters (mean duration and prevalence of OT3, and prevalence of CT3), while others can modulate only a subset of these parameters. The number of binding sites mediating these effects is unclear at the moment. Some electrophysiological and molecular biological data support a single site, other findings are best accounted for by the presence of multiple sites.

## Figures and Tables

**Table 1.** Characteristics of the Two Modes of Activity in the Presence of the Steroid Analog 11-TFMPD3α5αP

Origin of Activity	OT3 (ms)	Fraction OT3 (%)	Fraction CT3 (%)
50 µM GABA	7 ± 3	13 ± 7	27 ± 6
GABA + steroid (low Po episodes)	5±1	19±24	22±5
GABA + steroid (high Po episodes)	20 ± 3	32 ± 6	4 ± 2

The table shows the mean duration and prevalence (fraction) of the longest-lived intracluster open time component (OT3), and the prevalence of the longest-lived intracluster closed time component (CT3) under control conditions (GABA only) and in the presence of the steroid 11-TFMPD3α5αP. The control data are from Li *et al*., 2007b. Single-channel clusters in the presence of 50µ GABA and 50-1000 nM steroid (n = 4 patches) were broken into high and low Po episodes by eye, and the data from the two classes of episodes were analyzed separately.
